# Up-Regulation of HSFA2c and HSPs by ABA Contributing to Improved Heat Tolerance in Tall Fescue and Arabidopsis

**DOI:** 10.3390/ijms18091981

**Published:** 2017-09-15

**Authors:** Xiuyun Wang, Lili Zhuang, Yi Shi, Bingru Huang

**Affiliations:** 1 College of Agro-grassland Science, Nanjing Agricultural University, Nanjing 210095, China; wxy.550@163.com (X.W.); zhuanglili2001@163.com (L.Z.); 2 Key Laboratory of Grassland Ecosystem, College of Grassland Science, Gansu Agricultural University, Lanzhou 730070, China; shiyi214@126.com; 3 Department of Plant Biology and Pathology, Rutgers, the State University of New Jersey, New Brunswick, NJ 08901, USA

**Keywords:** tall fescue, abscisic acid, heat stress transcription factor, heat shock protein, heat tolerance

## Abstract

Abscisic acid (ABA) is known to play roles in regulating plant tolerance to various abiotic stresses, but whether ABA’s effects on heat tolerance are associated with its regulation of heat stress transcription factors (HSFs) and heat shock proteins (HSPs) is not well documented. The objective of this study was to determine whether improved heat tolerance of tall fescue (*Festuca arundinacea* Schreb.) by ABA was through the regulation of HSFs and HSPs. ABA-responsive transcriptional factors, ABA-responsive element binding protein 3 (FaAREB3) and dehydration-responsive element binding protein 2A (FaDREB2A) of tall fescue, were able to bind to the *cis*-elements in the promoter of tall fescue heat stress transcription factor A2c (Fa*HSFA2c*). Exogenous ABA (5 μM) application enhanced heat tolerance of tall fescue, as manifested by increased leaf photochemical efficiency and membrane stability under heat stress (37/32 °C, day/night). The expression levels of *FaHSFA2c*, several tall fescue *HSPs* (*FaHSPs*), and ABA-responsive transcriptional factors were up-regulated in plants treated with ABA. Deficiency of Arabidopsis heat stress transcription factor A2 (*AtHSFA2*) suppressed ABA-induction of *AtHSPs* expression and ABA-improved heat tolerance in Arabidopsis. These results suggested that HSFA2 plays an important role in ABA-mediated plant heat tolerance, and FaAREB3 and FaDREB2A may function as upstream *trans*-acting factors and regulate transcriptional activity of *FaHSFA2c* and the downstream *FaHSPs*, leading to improved heat tolerance.

## 1. Introduction

Abscisic acid (ABA) is known to play important roles in regulating plant responses to various abiotic stresses, especially those stresses involving dehydration, such as drought, salinity, and cold stress [1]. Some studies have also reported improved heat tolerance with ABA through exogenous application or manipulation of ABA-related genes [2,3,4]. Both ABA biosynthesis mutants (*aba* mutants) and ABA-insensitive mutants (*abi1* and *2*) showed defects in heat stress tolerance [5]. ABA may play roles in heat acclimation-induced heat tolerance or acquired heat tolerance [3]. Few studies have reported that the induced heat tolerance by ABA may be associated with enhanced osmotic adjustment due to the accumulation of osmoprotectants [6] and stimulation of antioxidant protection systems [7,8]. ABA-induced heat tolerance may involve the adjustment of multiple metabolic processes; however, how ABA may interact with molecular factors controlling heat tolerance is not well understood.

One of the major pathways of heat tolerance is through the activation or induction of heat-protective genes and proteins, including heat stress transcription factors (HSFs) and the downstream heat shock proteins (HSPs) [9,10]. HSFs play major roles in the transcriptional regulation of plant heat tolerance [11,12]. Among the large family of plant HSFs, the A2 subgroup of heat stress transcription factors (HSFA2s) belong to the most strongly induced proteins in plants exposed to heat stress and are key factors in the regulation network of heat responses [13,14]. It was reported that *HSFA2*-knockout plants became heat sensitive and showed reduced expression of many heat-related genes, which could be rescued by introducing a wild-type copy of *HSFA2* [15]. In addition, rice (*Oryza sativa*) heat stress transcription factor A2e (*OsHSFA2e*) or Arabidopsis heat stress transcription factor A2 (*AtHSFA2*) overexpression in Arabidopsis resulted in the up-regulation of HSPs, leading to enhanced heat tolerance [16,17]. HSPs, including small HSPs, HSP70, HSP90, and HSP101 are known to act as molecular chaperones, preventing misfolding and aggregation of other proteins involved in important physiological functions for plant adaptation to heat stress [12,18]. ABA was found to induce HSPs accumulation, conferring heat tolerance in some plant species [19,20,21]. The question of whether ABA-induced heat tolerance could be due to its interaction or regulation of *HSFA2* and *HSP* genes is yet to be addressed.

Regulatory sequences conferring ABA inducibility for stress responses include ABA response elements (ABREs) recognized by basic leucine zipper (bZIP) transcription factor family members, drought response elements (DREs) bound by APETALA2 (AP2) family proteins, RY/Sph element bound by B3 domain proteins, and binding sites for MYB- and MYC-class transcription factors [22]. ABRE binding factors (AREBs), DRE binding factors (DREBs), MYCs and MYBs function as transcriptional activators in ABA-responsive gene expression under drought or salinity stress [23,24,25]. However, which ABA-responsive genes and how those genes involved in regulating HSFs and HSPs improved heat tolerance are not clear.

Our previous studies have cloned heat stress transcription factor A2c (*FaHSFA2c*) from tall fescue (*Festuca arundinacea* Schreb.), and overexpression of *FaHSFA2c* resulted in improved heat tolerance in tall fescue and Arabidopsis [26]. The objectives of this study were to examine whether ABA may induce or up-regulate the expression of *FaHSFA2c* through direct binding of ABA respond genes to ABA responsive *cis*-elements in the promoter of *FaHSFA2c* and lead to the induction or up-regulation of downstream *HSP* genes, and to determine whether ABA-induced heat tolerance is associated with the interaction of ABA and *FaHSFA2c* and *FaHSPs*.

## 2. Results and Discussion

### 2.1. ABA-Responsive Factors of Tall Fescue Binding to Cis-Elements in FaHSFA2c Promoter

**Figure 1:**
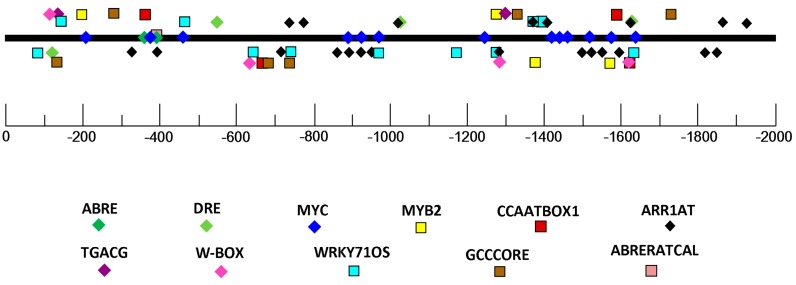
Predicted *cis*-elements on the *FaHSFA2c* promoter of tall fescue. The bold black line represents the promoter and the colorful icons represent different *cis*-elements and their positions on the promoter. Promoter sequences upstream of the ATG codon are marked by negative numbers.

As described in the introduction, how ABA may interact with HSFs is not well understood. *Cis*-elements in promoters conferring inducibility of gene expression by upstream regulatory factors are the connection points linking responsive factors and downstream genes [27]. Therefore, in order to determine regulatory factors for a target gene, one approach is to find *cis*-elements in the promoter of target genes that are related to the regulatory genes [28]. In this study, the promoter sequence with 2333 bp length (GenBank accession number: KX852425), upstream of the ATG codon of *FaHSFA2c* gene, was isolated from tall fescue genomic DNA. This sequence has 91% and 85% similarity to that of *Brachypodium distachyon* and *Aegilops tauschii HSFA2c*, respectively. *Cis*-elements on the 2000 bp length of promoter sequence upstream of the ATG codon of *FaHSFA2c* were predicted by using PLACE database [29]. Figure 1 shows the presence of five ABA-responsive *cis*-elements (ABRE, ABRERATCAL, DRE, MYB2, and MYC), two salicylic acid-responsive *cis*-elements (TGACG and W-BOX), one gibberellin-responsive *cis*-element (WRKY710S), and one ethylene-responsive *cis*-element (GCCCORE). The presence of multiple ABA-responsive *cis*-elements across the *FaHSFA2c* promoter suggested that ABA could be potentially able to induce or activate the expression of *FaHSFA2c*.

**Figure 2:**
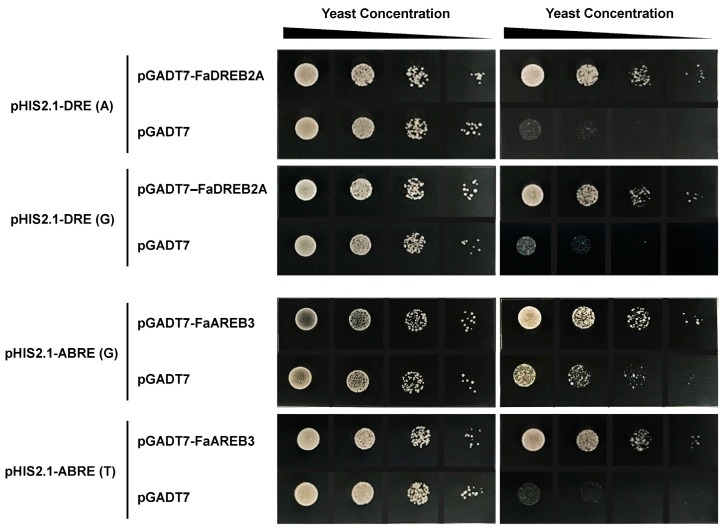
Binding activity of tall fescue dehydration-responsive element binding protein 2A (FaDREB2A) and ABA-responsive element binding protein 3 (FaAREB3) to the *cis*-elements from *FaHSFA2c* promoter of tall fescue. DRE (A), ACCGAC; DRE (G), GCCGAC; ABRE (G), ACGTGGC; ABRE (T), ACGTGTC.

The yeast one-hybrid assay is an effective approach to determine gene binding activities [30]. In order to investigate whether ABA activation of *FaHSFA2c* is by direct binding of ABA-responsive factors (FaAREB3 and FaDREB2A of tall fescue) to the promoter of *FaHSFA2c*, we tested the binding activity of the two proteins to the *cis*-elements in the *FaHSFA2c* promoter using the yeast system. The yeast one-hybrid results revealed that yeast cells co-transformed with FaDREB2A with a DRE element (ACCGAC or GCCGAC) or FaAREB3 with an ABRE element (ACGTGGC or ACGTGTC) were maintained viable in SD-WLH media with or without 3-AT supplements, compared with the negative control (Figure 2). It indicated that FaDREB2A and FaAREB3 were able to bind DRE and ABRE *cis*-elements in the *FaHSFA2c* promoter, respectively. It was reported that some heat-related genes that harbored DRE motifs in their promoter regions were suggested to be regulated directly by DREB2A, which is downstream of the HSFA1s in heat responses [31]. *FaHSFA2c* promoter contains both ABA responsive elements and heat shock element (HSE), and the gene expression might be regulated directly by ABA responsive factors in addition to *FaHSFA1*. AtHSFA6b was also reported to play important roles in the ABA-mediated heat stress, and AtHSFA6b directly bound to the promoter of *AtDREB2A* and enhanced its expression in Arabidopsis [2]. Therefore, FaDREB2A may be a crucial factor involved in crosstalk of ABA and heat responses. Our study first demonstrated that ABA may directly regulate *FaHSFA2c*, and FaDREB2A and FaAREB3 may function as upstream *trans*-acting factors of *FaHSFA2c*, regulating its transcriptional activity.

### 2.2. ABA Induced Gene Expression of FaHSFA2c and FaHSPs and Enhanced Heat Tolerance in Tall Fescue

**Figure 3:**
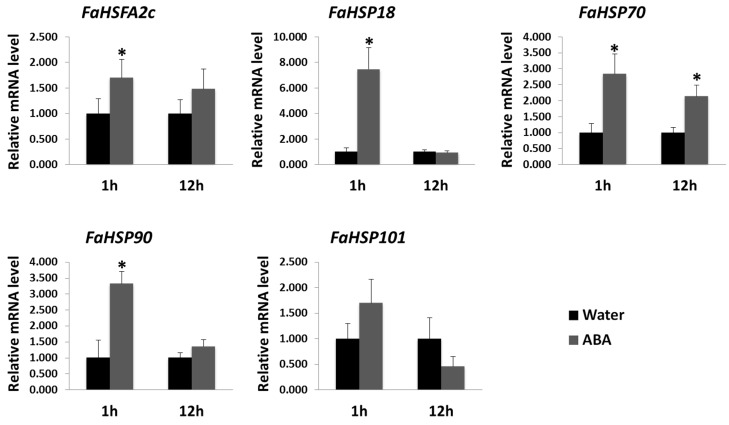
Gene expression of *FaHSFA2c* and *FaHSPs* in tall fescue by abscisic acid (ABA) induction, with water as control. Transcripts were measured at 1 and 12 h after treatments using quantitative real time polymerase chain reaction (qRT-PCR) and the relative mRNA level of ABA-treated plants was expressed as a fold of water control. Data are expressed as the mean values ± standard deviation (SD) of three biological replicates. Asterisk indicates significant difference between ABA and water treatments according to Student’s *t*-test at a significance level of 0.05.

The regulation of other *HSF* genes by ABA related to improved stress tolerance for salinity or drought has been documented [2,32], but its regulation of *HSFA2c* for heat responses has not been previously reported. To determine whether ABA could induce the expression of *FaHSFA2c*, the transcription levels of *FaHSFA2c* in tall fescue leaves were analyzed at 1 and 12 h after foliar application of ABA. The qRT-PCR results demonstrated that gene expression levels of *FaHSFA2c* were elevated within 1 h of ABA treatment (Figure 3). Transcripts of several *HSPs* were also up-regulated significantly at 1 h of ABA application for *FaHSP18* and *FaHSP90*, and at 1 and 12 h for *FaHSP70*. The expression of *FaHSP101* was not affected significantly by ABA either at 1 or 12 h. The induction and up-regulation of *FaHSFA2c* and downstream *HSP* genes, *FaHSP18*, *FaHSP90*, and *FaHSP70* by ABA treatment suggested that ABA is likely to play roles in regulating the heat-protection pathways in tall fescue.

**Figure 4:**
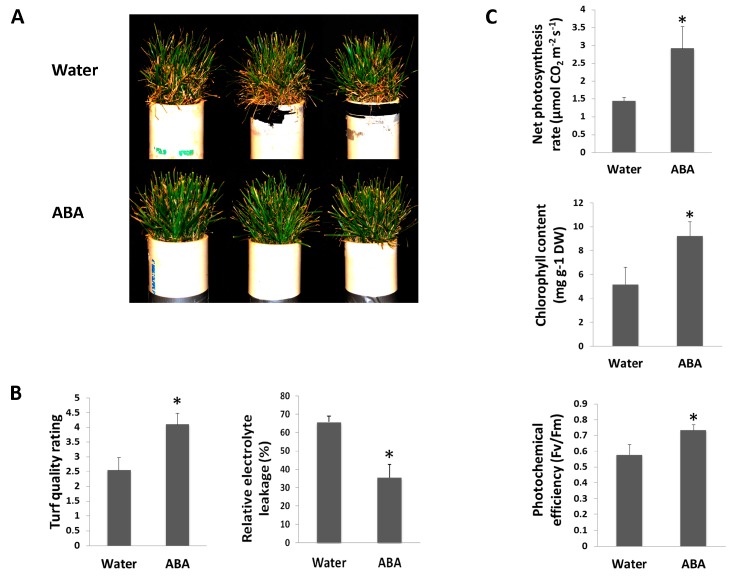
Effects of exogenous ABA on heat tolerance of tall fescue. (**A**) Phenotype; (**B**) turf quality and relative electrolyte leakage; (**C**) net photosynthesis rate, chlorophyll content, and photochemical efficiency of tall fescue plants with ABA or water treatment by 35 days of heat stress (37/32 °C). Three pots represent three biological replicates (*n* = 3) for each treatment. Asterisk indicates significant difference between ABA and water treatments according to Student’s *t*-test at a significance level of 0.05.

Heat tolerance evaluated by whole-plant performance or turf quality is positively correlated to physiological traits, such as membrane stability expressed as the reciprocal of electrolyte leakage, net photosynthetic rate, chlorophyll content, and photochemical efficiency in cool-season grass species [33]. In order to determine the physiological effects of ABA on heat tolerance of tall fescue, plants were foliar applied with ABA or water before and during heat stress. ABA-treated plants maintained significantly higher turf quality than the water control during prolonged periods of heat stress (35 days) (Figure 4A,B). Electrolyte leakage (EL) of leaves was evaluated as an indicator of membrane stability. EL values of ABA- and water-treated plants were 65.7% and 35.5%, respectively, indicating more stable membrane activity with ABA treatment under heat stress (Figure 4B). Foliar application of ABA maintained significantly higher net photosynthesis rate, chlorophyll content and photochemical efficiency compared to the water control by 35 days of heat stress (Figure 4C). Application of ABA was proven to be effective in mitigating heat-induced damage of membrane and photosynthesis systems, and ABA played positive roles in conferring the heat tolerance of tall fescue.

### 2.3. ABA Enhanced Transcription Levels of Stress-Responsive Genes in Tall Fescue under Heat Stress

**Figure 5:**
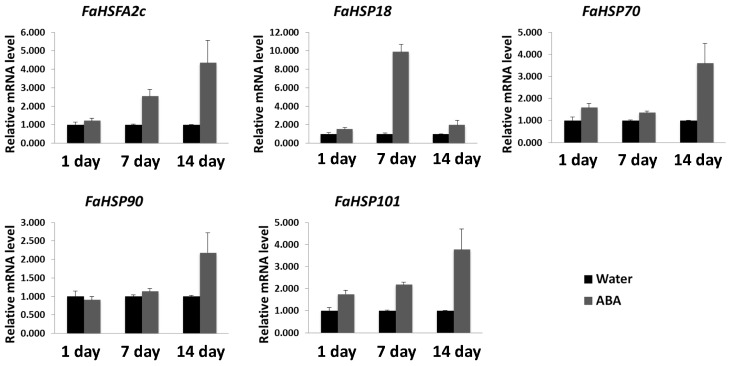
Gene expression analyses of *FaHSFA2c* and *FaHSPs* in tall fescue plants with ABA or water treatment during 14 days of heat stress. Transcripts were measured using qRT-PCR and the relative mRNA level of ABA treatment was expressed as a fold of water control at different time points. Data are expressed as the mean values ± SD of three biological replicates.

**Figure 6:**
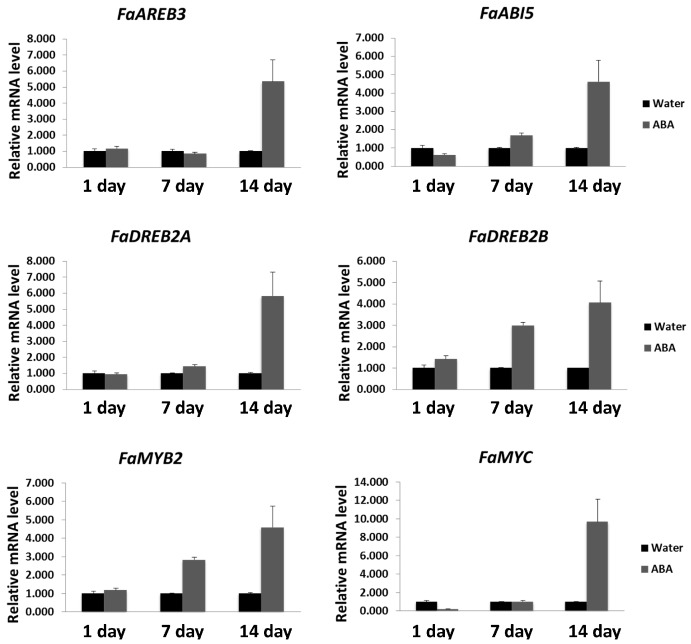
Gene expression analyses of *FaDREB2A*, *FaDREB2B*, *FaAREB3*, *FaABI5*, *FaMYB2*, and *FaMYC* in tall fescue plants with ABA or water treatment during 14 days of heat stress. Transcripts were measured using qRT-PCR and the relative mRNA level of ABA treatment was expressed as a fold change of the water control at different time points. Data are expressed as the mean values ± SD of three biological replicates.

As described above, ABA induced the expression of *FaHSFA2c* and *FaHSPs* even under normal temperature. We hypothesized that the improved heat tolerance by ABA, as manifested by the physiological changes, may be due to the activation of *FaHSFA2c* and *FaHSPs* under heat stress. To test this hypothesis, transcription levels of stress-responsive genes were analyzed in ABA-treated and untreated plants exposed to heat stress. ABA enhanced the transcription levels of *FaHSFA2c* and *FaHSPs* (*FaHSP18*, *FaHSP70*, *FaHSP90*, and *FaHSP101*) by 14 days of heat stress (Figure 5). To further characterize gene expression patterns of the potential binding transcription factors of *FaHSFA2c*, we performed qRT-PCR analyses for *FaDREB2A*, *FaDREB2B*, *FaAREB3* and its homolog *FaABI5*, *FaMYB2*, and *FaMYC* (Figure 6). These genes also had different degrees of transcriptional increases with ABA treatment within 14 days of heat stress. The results demonstrated that ABA enhanced transcription of these stress-responsive genes under heat treatment, which may result in the improved heat tolerance in tall fescue with ABA treatment.

DREB2A was reported to activate the *HSFA3* promoter to enhance its transcriptional activity during heat stress [34], and could bind the DRE elements on the *FaHSFA2c* promoter in our study. Therefore, DREB2A could activate heat-inducible target genes by binding their DRE elements [35]. During heat stress, heat-responsive factors DNA polymerase II subunit B3-1 (DPB3-1) and nuclear factor Y (NF-Y) B3 subfamily (NF-YB3) form a trimer with NF-YA2 to enhance transcriptional activity of DREB2A to activate heat stress inducible target genes [35]. It can be inferred that DERB2A may act as an upstream regulatory factor in the HSF network controlling heat tolerance. The direct regulatory functions of other ABA-response elements, *DREB2B*, *AREB3*, *ABI5*, *MYB2*, and *MYC* in expression of *HSFA2c* and other *HSFs* have not been previously reported, which deserve further investigation.

### 2.4. Diminished Heat Tolerance in Arabidopsis Mutants of AtHSFA2 Partially Rescued by ABA

**Figure 7:**
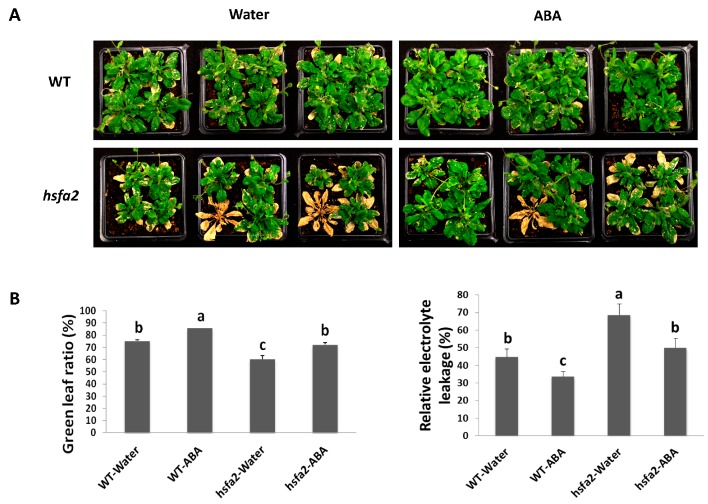
Heat tolerance of Arabidopsis WT and *hsfa2* mutant treated with ABA. (**A**) Phenotype; (**B**) green leaf ratio and relative electrolyte leakage of wild-type (WT) and *hsfa2* mutant plants treated with ABA or water after 3 days of recovery following 2 weeks of heat stress (37/32 °C). Three pots represent three biological replicates (*n* = 3) for each treatment. Letters above columns indicate significant difference among the data according to protected LSD test at a significance level of 0.05.

Mutants with the knockdown of target genes are excellent plant materials for the confirmation of gene functions and regulations [36]. For further investigation of the interactive effects of ABA and *HSFA2* on heat tolerance, Arabidopsis *hsfa2* mutant was treated with ABA or water and then exposed to heat stress (Figure 7A). The green leaf ratio, as an indicator of leaf senescence, decreased significantly in *hsfa2* mutant plants exposed to heat stress, which was significantly lower than that of the wild type (WT). ABA treatment caused increases in the green leaf ratio in *hsfa2* plants, but did not result in full recovery to the level of the WT. EL, as an indicator of membrane stability, increased significantly in *hsfa2* mutant plants exposed to heat stress, which was significantly greater than that of the WT plants. With ABA treatment, EL decreased in *hsfa2* plants, but did not decrease to the level of the WT (Figure 7B). These results suggest that *AtHSFA2* could play critical roles in regulating plant tolerance to heat stress and ABA could partially rescue the phenotypes of leaf senescence and membrane damage. Deficiency of *AtHSFA2* negatively affected ABA-mediated heat tolerance.

**Figure 8:**
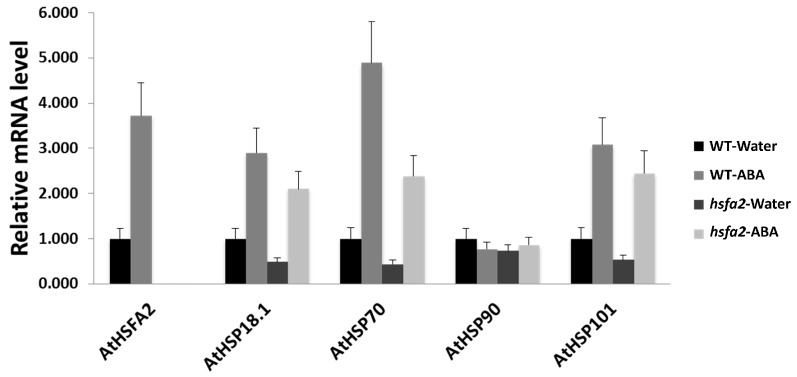
Gene expressions of *AtHSFA2* and *AtHSPs* in Arabidopsis WT and *hsfa2* plants pretreated with ABA or water after 1 h of heat treatment. Transcripts were measured using qRT-PCR and the relative mRNA levels were expressed as a fold of water-treated WT for each gene. Data are expressed as the mean values ± SD of three biological replicates.

**Figure 9:**
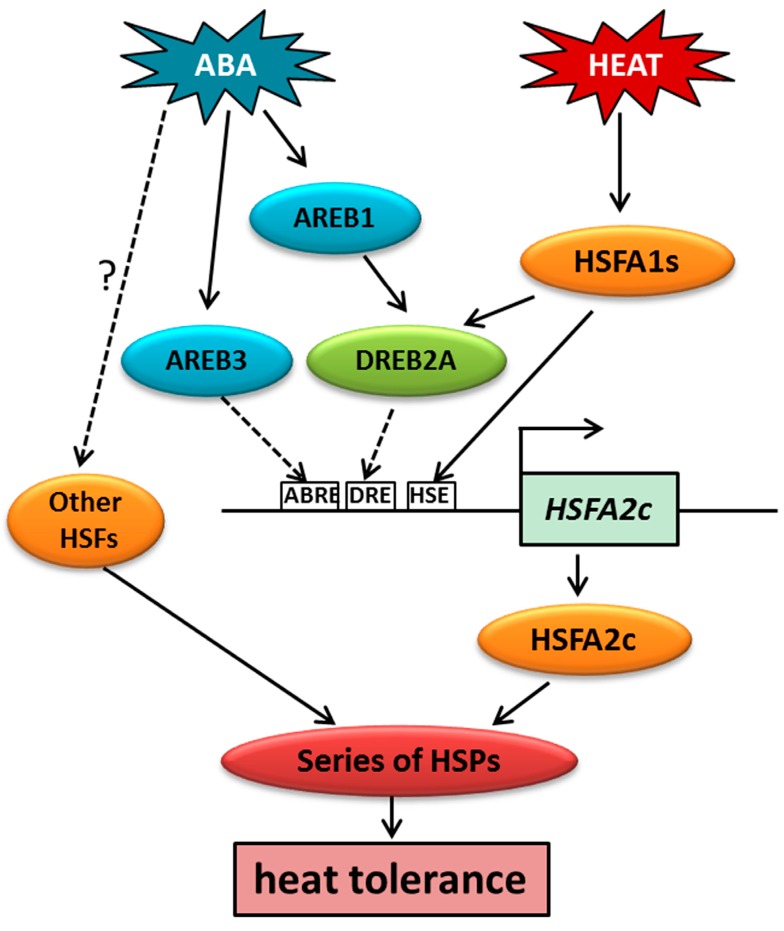
Transcriptional regulation of heat stress transcription factors (HSFs) and heat shock proteins (HSPs) by ABA-responsive transcription factors in heat response. Under heat stress, HSFA1s could induce expression of DREB2A, which is also regulated by ABA-responsive factor AREB1. DREB2A and AREB3, in addition to HSFA1s, may act as transcriptional regulator upstream of HSFA2c; further enhancing expression of series of HSPs. ABA may also regulate expression of HSPs by inducing other HSFs. HSPs act as molecular chaperons for other proteins and enhanced heat tolerance in heat stress. Dashed lines denote links to be confirmed.

In order to determine whether phenotypic changes in *hsfa2* mutants and ABA treated plants were associated with the expression of *AtHSFs* and *AtHSPs*, qRT-PCR was performed after heat treatment (Figure 8). Plants of *hsfa2* mutant exhibited significantly lower levels of expression for *AtHSP18.1*, *AtHSP70*, and *AtHSP101* than WT under heat stress. The expression of *AtHSFA2* was up-regulated by ABA in the WT, whereas this gene was not detected in its knockout mutant *hsfa2*. ABA caused up-regulation of *AtHSP18.1*, *AtHSP70*, and *AtHSP101* in WT and *hsfa2* plants, but the expression levels of those genes were significantly lower in ABA-treated mutants than in the WT. These results demonstrated that deficiency of *AtHSFA2* suppressed ABA-induction of *AtHSPs* expression. It is worth noting that ABA had only partially rescued *AtHSFA2*-related phenotypes and gene expression, suggesting that other ABA-responsive factors in addition to *AtHSFA2* are involved in mediating heat tolerance (Figure 9). ABA may also affect heat tolerance through regulating other processes, such as antioxidant metabolism [7,8] and osmotic protection [6]. It is known that multiple HSFs play roles in heat responses in Arabidopsis [11,37]. The interactive effects of ABA with other *HSFs* besides *HSFA2* should be further explored.

## 3. Materials and Methods

### 3.1. Plant Materials and Growth Conditions

Tall fescue cv. “Kentucky 31” seeds were sown in plastic pots (10 cm diameter and 20 cm depth) filled with loamy soil in a greenhouse on 18 January 2016, with natural sunlight and average temperature of 23/18 °C (day/night) for three-week seedling establishment. On 13 February, three-week old seedlings were transferred to growth chambers controlled at 22/17 °C with 60% relative humidity and 12-h photoperiod with 650 μmol·m^−2^·s^−1^ photosynthetically active radiation for 7 days prior to ABA and heat stress treatments. During the seedling establishment period, plants were trimmed every three days to maintain canopy height at about 8 cm, irrigated every two days, and fertilized weekly with Hoagland’s nutrient solution [38].

Arabidopsis ecotype Col-0 was used as the WT, and the HSFA2 (At2g26150) knockout mutant *hsfa2* (SALK_008978) [36] was obtained from the Arabidopsis Biological Resource Center (ABRC; Ohio State University). Sterilized seeds of WT and *hsfa2* mutant were geminated in Murashige and Skoog (MS) medium and 5-day-old seedlings were transferred into plastic pots filled with loamy soil. These plants were maintained in a growth chamber at 25/20 °C with 65% relative humidity and 10-h photoperiod with 150 μmol·m^−2^·s^−1^ photosynthetically active radiation before treatments.

### 3.2. ABA and Heat Treatments

To examine gene expression of *FaHSFA2c* and *FaHSPs* as affected by ABA, 4-week-old tall fescue plants were foliar sprayed with 5 μM ABA on February 20, and leaves were collected at 1 and 12 h after ABA treatment. To investigate the effects of ABA on heat tolerance, 4-week-old plants were foliar sprayed with 5 μM ABA or water every two days for three times from February 20 to 24, prior to exposure of heat stress (37/32 °C, day/night temperature) and sprayed once every week during the 35 days of heat treatment. The control temperature is 22/17 °C. Five micromolar ABA was used in this study because a preliminary screening test of different concentrations of ABA (0.2, 1, 5, 25, and 50 μM) demonstrated that this was the optimal concentration for the induction of heat tolerance in tall fescue. Heat treatments for tall fescue were repeated in four growth chambers (37/32 °C, day/night temperature). The ABA and water control treatment had three biological replicates (pots), which were placed randomly inside each of four growth chambers.

One-month-old WT and *hsfa2* Arabidopsis plants treated with ABA or water were exposed to heat stress (37/32 °C) for two weeks and recovery at normal temperature for 3 days. The treatments were repeated in four growth chambers. The method of ABA application for Arabidopsis was same as for tall fescue. Leaves of three replicates (pots) were collected at 1 h after heat treatment for gene expression analysis or at the end of recovery for physiological measurements.

### 3.3. FaHSFA2c Promoter Isolation and Cis-Elements Analysis

The *FaHSFA2c* promoter was isolated from tall fescue genome DNA with nested-PCR by using Universal GenomeWalker Kit (Clontech Laboratories Inc., Mountain View, CA, USA) based on the known sequence. The promoter sequence was verified by sequencing and BLAST search [39] on the website of NCBI (National Center for Biotechnology Information, available online: http://www.ncbi.nlm.nih.gov/). The Plant cis-acting regulatory DNA elements (PLACE) database (Available online: http://www.dna.affrc.go.jp/htdocs/PLACE/) [29] was used to predict *cis*-elements on the promoter.

### 3.4. Y1H Assay

The tall fescue *FaAREB3* and *FaDREB2A* open reading frame (ORF) sequences were PCR amplified from leaf cDNA using primers with *Sma* I and *Xho* I restriction sites (Supplemental Table S1) and inserted at the corresponding restriction sites of pGADT7 vector. Three tandem repeats of DRE (ACCGAC or GCCGAC) or ABRE (ACGTGGC or ACGTGTC) were introduced into a pHIS2.1 vector with *EcoR* I and *Spe* I restriction sites and confirmed by sequencing with pHIS2.1 primers. Constructed pGADT7 and pHIS2.1 plasmids were co-transformed into Y187 yeast competent cells by using Frozen-EZ Yeast transformation II kit (Zymo Research, Irvine, CA, USA). Binding activity was determined by colony growth on SD medium lacking tryptophan, leucine, and histidine (SD-WLH) and 50 mM 3-amino-1,2,4-triazole (3-AT) was used to reduce background interference. 

### 3.5. Gene Expression Analysis

RNA of tall fescue or Arabidopsis leaves was extracted using TRIzol reagent (Invitrogen, Carlsbad, CA, USA) and cDNA was synthesized with PrimeScript RT reagent Kit with gDNA Eraser (Perfect Real Time) (Takara, Otsu, Japan). Real time quantitative polymerase chain reaction (qRT-PCR) was performed on a Step One Plus Real-Time PCR System (Applied Biosystems, Foster City, CA, USA) with Power SYBR Green PCR Master Mix (Applied Biosystems). *AtActin2* and *Faα-Tubulin* were applied as the reference gene for Arabidopsis and tall fescue, respectively. Data were normalized according to the reference gene expression levels and determined by 2^−ΔΔ*C*t^ calculation methods. Primers for this study were listed in Table S1.

### 3.6. Physiological Measurements

Physiological measurements of tall fescue were performed at 35 days of heat treatment on March 26. Turf quality was visually rated on a scale of 1–9, with 9 indicating the best in all quality components and 1 indicating completely brown, dead turf, based on color, density, and uniformity of grass canopy [40]. Leaf net photosynthetic rate was measured using a LI-6400 portable photosynthesis system (LI-COR, Lincoln, NE, USA) and photochemical efficiency was determined with a pulse-modulated fluorometer Fim 1500 (Analytical Development Company Ltd., Hoddesdon, UK). Chlorophyll content was extracted with soaking leaf tissue in dimethyl-sulfoxide [33]. Relative electrolyte leakage was determined using a reported method [41]. The green leaf ratio was measured as the number of green leaves (that more than half of a leave maintaining green) to total number of leaves per pots of plants.

### 3.7. Statistic Analysis

Physiological and gene expression data were analyzed using the analysis of variance (ANOVA). Means of treatment effects with ABA and heat stress were compared using the protected least significance test (LSD) or Student’s *t*-test at a significance level of 0.05 with SPSS statistical program (IBM Corporation, Armonk, NY, USA).

## 4. Conclusions

The expression levels of *FaHSFA2c*, several *FaHSPs*, and ABA-responsive transcriptional factors were up-regulated in tall fescue plants with ABA treatment under heat stress. ABA-responsive transcriptional factors, FaAREB3 and FaDREB2A, were able to bind to the *cis*-elements in *FaHSFA2c* promoter. Exogenous ABA application enhanced heat tolerance of tall fescue, as manifested by increased leaf photochemical efficiency and membrane stability under heat stress. Deficiency of *AtHSFA2* suppressed ABA-induction of *AtHSPs* expression and ABA-improved heat tolerance in Arabidopsis. Those results suggested that FaAREB3 and FaDREB2A might function as upstream *trans*-acting factors and regulate transcriptional activity of *FaHSFA2c* and the downstream *FaHSPs*, leading to improved heat tolerance in tall fescue. The investigation of the direct regulatory function of other ABA-response elements, *DREB2B*, *AREB3*, *ABI5*, *MYB2*, and *MYC* in the expression of *HSFA2c* and other *HSFs*, as well as downstream *HSPs* would help provide further insights into the molecular regulatory networks associated with heat stress protection involving HSFs and HSPs.

## Supplementary Materials

Supplementary materials can be found at www.mdpi.com/1422-0067/18/9/1981/s1.
