# Supplementary material for: Up-Regulation of HSFA2c and HSPs by ABA Contributing to Improved Heat Tolerance in Tall Fescue and Arabidopsis
Source: Int J Mol Sci. 2017 Sep 15;18(9):1981. doi: 10.3390/ijms18091981 (PMC5618630; doi:10.3390/ijms18091981)
Supplement: Supplementary file 1 [file ijms-18-01981-s001.pdf]

Supplemental material

Table S1. Primers used in this study.

**Primer Name**	**Forward Primer Sequence (5'-3')**	**Reverse Primer Sequence (5'-3')**
Faα-Tubulin	ATGCTTTCGTCTTATGCCC	CTCTTGGTTTTGATGGTTGC
FaHsfA2c	TGTTGTTTGATTCGGGTGTCC	ATCCATCTTTTCCTCATCTCTTTTAC
FaHsp18	TGTCTCCTTCCAGTCCATACGA	CTTCATTTTCCCCTCGCATAGA
FaHsp70	ACTTGCCCAGCAGATTGTTGT	ACCACCATCCCGACCAAGAA
FaHsp90	TCTGAAGATAGATGACAGCGAGG	GCACGGAGAGTCCACCACAC
FaHsp101	GAGGGTGAGACTGGTGAGAACTT	GCTTCATACTGTCCAACCACCC
FaAREB3	CTCCTGGACACTCCTTTTCGGT	TTCCTGATGACTTGGTGCCTGA
FaABI5	AGGACTTGAACGAGGAACTGG	GCTATTTTGAGCATCACCAGG
FaDREB2A	CATCAAGAAGTGGAAGGAGCAGAA	ATCTCAGCCACCCATTTGCC
FaDREB2B	GCCAGTCTACCACAACATCTAA	ACCTCACTGGACTTGAAATCTC
FaMYB2	CCGAGTATTTCTGCGTTTGACA	GAGAACTTTGAGGAAGCCCAGG
FaMYC	TCATCCATCCTTCCTCCTAATAGAC	AGCAGCCAGACATCCAGAACTC
pHIS2.1	GCCTTCGTTTATCTTGCCTGCTC	CGATCGGTGCGGGCCTCTTC
FaAREB3-ORF	cccgggATAGGCCTTTGCCTGCT	ctcgagCCAATTCAGAAAGCCGCT
FaDREB2A-ORF	cccgggATTGCCTTGATGTCCAGG	ctcgagCGTGACTACAACCCTCAT
DRE (A)	aattcACCGACACCGACACCGACa	ctagtGTCGGTGTCGGTGTCGGTg
DRE (G)	aattcGCCGACGCCGACGCCGACa	ctagtGTCGGCGTCGGCGTCGGCg
ABRE (G)	aattcACGTGGCACGTGGCACGTGGCa	ctagtGCCACGTGCCACGTGCCACGTg
ABRE (T)	aattcACGTGTCACGTGTCACGTGTCa	ctagtGACACGTGACACGTGACACGTg
AtActin2	TGCCAATCTACGAGGGTTTC	TTCTCGATGGAAGAGCTGGT
AtHsfA2	ACACATCTACTTGTTGCGGTCTAAG	CGAAATCAGAGTTTTTTATTCATCAG
AtHSP18.1	GGGAAGTTTATGAGAAGGTTTAGGTT	CAAGCCAAGAAAAAAACACAAACT
AtHsp70	GAAGAGGTGGATTAGAGCGTGTTTAGT	GACACAATACAAAGAAAAGACTCGCTA
AtHsp90	CGAAGAGGACGAACCAGTTAAG	GGCTAGTTCCCAATCCCAATAC
AtHsp101	AAAATGCAACCTTTTGGAGCC	TTCATAACCTCTGGACCTTTGAGAC
